# Gut–Liver Axis: How Do Gut Bacteria Influence the Liver?

**DOI:** 10.3390/medsci6030079

**Published:** 2018-09-17

**Authors:** Peter Christopher Konturek, Igor Alexander Harsch, Kathrin Konturek, Monic Schink, Thomas Konturek, Markus F. Neurath, Yurdaguel Zopf

**Affiliations:** 1 Department of Internal Medicine 2nd, Thuringia-Clinic Saalfeld, Teaching Hospital of the University of Jena, 68, D-07318 Jena, Germany; iharsch@thueringen-kliniken.de (I.A.H.); kathrin.konturek@web.de (K.K.); 2 1st Department of Internal Medicine, University Erlangen-Nuremberg, 91054 Erlangen, Germany; Monic.Schink@uk-erlangen.de (M.S.); Markus.Neurath@uk-erlangen.de (M.F.N.); Yurdaguel.Zopf@uk-erlangen.de (Y.Z.); 3 Department of Medicine, St. Elizabeth’s Medical Center, Tufts University School of Medicine, Boston, MA 02135, USA; konturek@me.com

**Keywords:** gut microbiota, dysbiosis, chronic liver diseases

## Abstract

Chronic liver diseases are a major cause of morbidity and mortality worldwide. Recently, gut dysbiosis was identified as an important factor in the pathogenesis of liver diseases. The relationship between gut microbiota and the liver is still not well understood; however, dysfunction of the gut mucosal barrier (“leaky gut”) and increased bacterial translocation into the liver via the gut–liver axis probably play crucial roles in liver disease development and progression. The liver is an important immunological organ, and, after exposure to gut-derived bacteria via portal circulation, it responds with activation of the innate and adaptive immune system, leading to hepatic injury. A better understanding of the pathophysiological links among gut dysbiosis, the integrity of the gut barrier, and the hepatic immune response to gut-derived factors is essential for the development of new therapies to treat chronic liver diseases.

## 1. Introduction

The gut microbiota forms a complex microbial community that has a major impact on human health [1]. The more than 100 trillion microorganisms in the gut show high metabolic activity and are in continuous dialogue with the host immune system [2]. Moreover, the gut microbiota is an important source of metabolites, hormones, and neuro-mediators that directly regulate gut function and indirectly modulate the function of extra-intestinal organs such as the liver, brain, and kidney [3]. 

The impact of the gut microbiota on human health and the physiology of the gastrointestinal tract is a rapidly evolving field of research. Multiple studies showed that a well-balanced intestinal flora is essential for health [4], implying that human health strongly depends on the composition and function of the gut microbiota. Interestingly, each human being has their individual characteristic composition of gut microbiota termed “microbial fingerprint” [5]. 

Multiple factors may influence microbiota composition and can increase the risk of dysbiosis, including diet, exposure to stress, the broad use of antibiotics, aging, and comorbid conditions [6].

Importantly, communication between the host and gut microbiota occurs bidirectionally. This interaction between the gut microbiota and host is especially apparent in the immune system. Gut microorganisms impact the development and function of the immune system. On the other hand, the host immune system (both innate and adaptive) shapes the microbiota composition and diversity in the gut.

There is strong evidence that gut microorganisms have an important impact on the physiology of the gastrointestinal tract itself and may also affect the function of extra-intestinal organs including the liver, kidney, or brain, the cardiovascular system, and/or the bone system. The communication with organs beyond the gastrointestinal tract (“extra-intestinal functions”) occurs directly via Toll-like receptors (TLR) and indirectly via different bacterial metabolites and signaling molecules [7].

In recent years, it became clear that the intestinal flora plays a crucial role in human physiology and is involved in the pathophysiology of chronic diseases within and outside the gut [8]. 

A well-balanced diet rich in fibers and unsaturated fats, like a “Mediterranean diet”, leads to an increase in anti-inflammatory bacterial taxa such as *Bifidobacteria* or *Akkermansia*. These bacteria provide important beneficial signals for the host immune system, and may be responsible for the development of immune tolerance to food. Moreover, these bacteria strengthen the gut barrier, and thus, prevent bacterial translocation through the gut wall and resulting endotoxemia. In contrast, the “western diet” contains food rich in saturated fats and carbohydrates leading to a significant increase in pro-inflammatory taxa such as *Bacteroides*. This switch in the gut microbiota composition may have detrimental effects on the gut barrier resulting in low-grade inflammation of the intestinal mucosa [9].

## 2. Gut–Liver Axis

From the physiological point of view, one of the most important links between gut microbiota and extra-intestinal organs is the gut–liver axis. It represents a close functional and bidirectional communication between the intestine and the liver. 

Since many years, it is well known that the liver is continually exposed not only to products of digestion and absorption, but also to gut-derived factors including bacteria and bacterial components like lipopolysaccharide (LPS). The venous system of the portal circulation defines the gut–liver axis and highlights the close anatomical and functional interaction of the gastrointestinal tract and the liver [10]. Physiologically, this axis seems to be an important operative unit that protects the host against potentially harmful and toxic substances from the gut, thereby maintaining the homeostasis of the immune system [11].

The portal vein is the direct venous outflow from the intestine. When the intestinal barrier is damaged and shows increased permeability, the liver is automatically exposed to numerous toxic factors derived from the intestine, as well as to intestinal bacteria. An existing intestinal dysbiosis would further amplify these processes [12].

A dysfunctional intestinal microbiota is associated with an increase in intestinal permeability, and consequently, an exposure of the liver to bacterial components termed pathogen-associated molecular patterns (PAMP) and damage-associated molecular patterns (DAMP); both can result in hepatic injury. PAMPs may act directly on hepatocytes or cells of the hepatic innate immune system such as Kupffer cells or stellate cells. The activated hepatic immune system induces pro-inflammatory pathways and may also influence antiviral and anti-apoptotic pathways in hepatocytes. These effects can be both detrimental (activation of immune response, release of pro-inflammatory cytokines) and beneficial (cytoprotection and regeneration of hepatocytes). It is important to mention that the liver not only receives microbial input, but conversely influences the intestinal microbes via bile acids and immunoglobulin A (IgA) antibodies, thereby playing an important regulatory role in the control of microbial populations [13].

## 3. Dysbiosis and Liver Diseases

**Figure 1:**
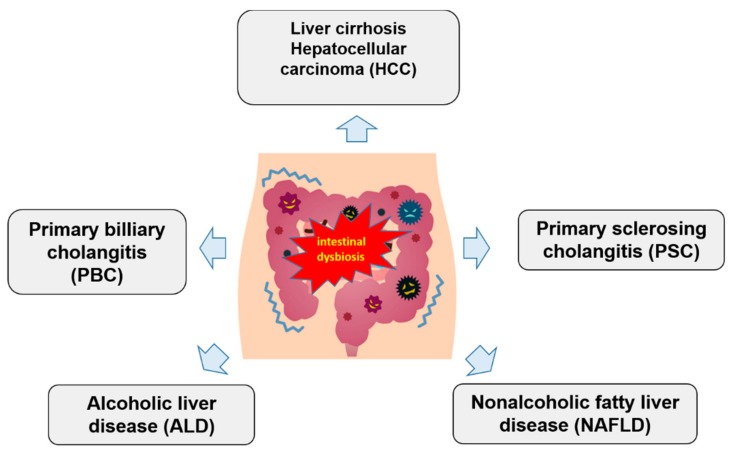
Schematic association between gut microbiota dysbiosis and chronic liver diseases.

Our understanding of liver diseases, especially liver cirrhosis, changed dramatically over the last decade with the introduction of culture-independent microbiome analysis. Experimental studies in animals and humans showed that many liver diseases are linked to intestinal dysbiosis (Figure 1). However, it is still not known if intestinal dysbiosis is a cause or a result of liver damage, the often cited “chicken-egg” problem [14]. 

In recent years, a continuous increase in the prevalence and the mortality of liver diseases was observed. The most common chronic liver diseases in western countries are alcoholic liver disease (ALD) and nonalcoholic fatty liver disease (NAFLD) that can progress to liver cirrhosis and/or hepatocellular carcinoma [15,16].

A better understanding of the pathophysiological connections among gut dysbiosis, the integrity of the gut barrier, and the hepatic immune response to gut-derived factors is crucial for the development of new therapies to treat chronic liver diseases or to at least prevent their progression and the development of complications. 

## 4. Gut Microbiota and Alcoholic Liver Disease

Excessive alcohol consumption is a leading cause of chronic liver disease worldwide [17]. The stages of ALD are hepatic steatosis, steatohepatitis, and ultimately, liver cirrhosis and/or hepatocellular carcinoma, often with an overlapping histology. It was suggested that not only the quantity of alcohol, but also the interplay with genetic host and/or environmental factors may be responsible for the development of hepatic injury in ALD [18].

Furthermore, experimental and clinical data imply that intestinal microbiota might also play an important role in the pathogenesis of ALD [19]. This potential link between gut bacteria and hepatic injury due to alcohol consumption was postulated more than 20 years ago. Adachi et al. showed for the first time that antibiotics prevent liver injury in rats following long-term exposure to ethanol [20]. Casafont Morencos et al. demonstrated the presence of excessive or abnormal microbiota in the small bowel (small intestinal bacterial overgrowth (SIBO)) in patients with alcoholic cirrhosis, and postulated its role in the pathogenesis of ALD [21]. The recent meta-analysis by Shah et al. further supports this observation showing higher SIBO prevalence in patients with chronic liver disease as compared to a healthy control [22]. In addition, an imbalance between native Firmicutes and Bacteroidetes species was described, with the former decreasing and the latter increasing [23].

The main characteristic of ALD is an increased gut permeability due to the direct toxic effect of alcohol on the epithelial cells in the gastrointestinal tract and the decreased expression of tight-junction proteins. This disruption of the intestinal barrier results in significant elevation of endotoxin plasma levels that may cause hepatic injury (“autotoxic concept”). Furthermore, it was reported that individual susceptibility to ALD may depend on the composition of the gut microbiota [24]. This hypothesis is supported by animal studies showing the acceleration of alcohol-induced inflammation in germ-free mice after transplantation of gut microbiota from alcoholic patients [25]. 

With the establishment of the dysfunctional intestinal microbiota as an important factor responsible for the onset and maintenance of ALD, different strategies to modulate gut microbiota were investigated in experimental and clinical studies. Most studies investigating the modulation of gut microbiota in ALD focused on the role of different probiotics in the treatment of this disease. The potential beneficial effects of probiotics on ALD include (1) quantitative and qualitative improvement of gut microbiota composition (increase in *Lactobacilli* and *Bifidobacteria*, increase in gut microbiota diversity); (2) improvement of liver function tests; (3) strengthening of gut-barrier permeability; (4) decrease in pro-inflammatory cytokines like tumor necrosis factor α (TNFα) and bacterial endotoxin levels in the blood; and (5) histologic improvement of liver steatosis and hepatic inflammation [26,27]. Modulation of the gut microbiota appears to be a promising therapeutic strategy in patients with ALD. Future therapy may employ engineered microbiota that decrease the permeability of the gut barrier and reduce the release of pro-inflammatory cytokines in the gut. Further clinical trials focusing on the role of gut microbiota in ALD are needed.

## 5. Gut Microbiota and Nonalcoholic Liver Disease

Nonalcoholic fatty liver disease represents the most common form of chronic liver disease that is closely linked to metabolic syndrome and increased insulin resistance. In recent years, both the incidence and prevalence of this disease substantially increased. NAFLD is recognized as a global public health problem [28]. It is a very common disease, and interestingly, it especially affects patients with obesity and diabetes type 2. NAFLD is characterized by the excessive accumulation of triglycerides in hepatocytes in the absence of alcohol consumption (defined as less than 20 g and 30 g per day in women and men, respectively). Similar to ALD, NAFLD may progress to steatohepatitis, liver cirrhosis, and even hepatocellular carcinoma [29].

Multiple risk factors contribute to the pathogenesis of NAFLD, among them are genetic and dietary factors, distribution of adipose tissue (in particular, the presence of visceral fat), and dysbiotic intestinal microbiota. Recent studies indicate a strong involvement of gut microbiota in the pathophysiology of NAFLD [30].

An underlying intestinal dysbiosis can cause hepatic steatohepatitis via the following pathophysiological events: (1) increase in hepatic inflammation leading to the development of steatohepatitis (due to metabolic entotoxemia and TLR-mediated cytokine production); (2) increase in insulin resistance; (3) hepatic de novo lipogenesis (steatosis); (4) change in bile-acid metabolism and farnesoid X receptor (FXR) signaling; (5) change in gut-barrier permeability (“leaky gut”) and induction of oxidative stress and inflammation by endogenous ethanol; and (6) decreased very-low-density lipoprotein (VLDL) assembly and secretion due to changed choline metabolism in the dysbiotic gut. Recent research indicates that compositional alterations of intestinal microbiota play a critical role in the development of NAFLD. Typical compositional changes observed in NAFLD are an increase in Bacteroidetes, a decrease in Firmucutes, and a rise in pro-inflammatory taxa such as Proteobacteria and Enterobacteriaceae [31].

Loss of barrier integrity due to dysbiosis leads to a consequent increase in bacterial translocation and to metabolic endotoxemia, which are key pathophysiological events for hepatic TLR system activation, and thus, for a local hepatic and systemic inflammatory response.

Gut-microbiota-targeted therapies in NAFLD include the use of probiotics, a collection of bacteria with beneficial effects on the host metabolism, and prebiotics, which are indigestible food ingredients that selectively stimulate the growth of anti-inflammatory taxa and suppress that of pro-inflammatory taxa. Recently, the effect of synbiotics, which are a combination of prebiotics and probiotics, on NAFLD was investigated [32,33]. Studies with probiotic-based therapy in NAFLD were mainly performed in animal models for this disease using high-fat diet (HFD)-induced fatty liver disease. Probiotic bacteria like *Lactobacillus* or *Bifidobacterium* demonstrated some promising effects, e.g., attenuation of hepatic fat accumulation, reduction of insulin resistance, limitation of oxidative and inflammatory liver damage, and decrease in serum lipids [34]. Small human studies showed a beneficial effect of probiotics on liver damage, along with a decrease in aminotransferases levels, and a reduction in total cholesterol and low-density lipoprotein cholesterol (LDL-C) concentrations [35]. Similarly, some prebiotics limited liver injury and reduced the levels of serum aminotrasfersases and insulin [36,37,38].

In the first randomized human study, Mofidi et al. showed positive effects of synbiotics on fibrosis and the serum level of aminotransferases [39]. A recent meta-analysis by Khalesi et al. found that probiotics and synbiotics given as supplements to nonalcoholic steatohepatitis (NASH) patients improve the serum concentration of liver enzymes [40].

## 6. Gut Microbiota and Immune-Mediated Liver Diseases

There is accumulating evidence that dysfunctional gut microbiota might be implicated in the pathogenesis of autoimmune diseases, primary billiary cirrhosis (PBC) and primary sclerosing cholangitis (PSC). These two clinical entities represent chronic cholestatic liver diseases mediated by the immune system. They are characterized by portal inflammation and slowly progress to liver fibrosis and cirrhosis.

Since many years, it is postulated that these diseases may be triggered by unknown environmental factors in genetically susceptible subjects. The gut microbiota could represent the missing link in these pathogenetic events. Potential causes/trigger for the development of PSC/PBC are (1) intestinal dysbiosis; (2) a change in bile-acid composition; (3) compositional alterations of billiary microbiota; and (4) diverse unfavorable bacterial products (PAMPs) and metabolites [41].

In a recent publication, Tang et al. demonstrated reduced microbial species richness and a distinct overall microbial diversity in PBC patients compared with healthy controls. PBC microbial dysbiosis was characterized by altered abundances of 12 genera, and the dysbiosis was partially reversed during ursodeoxycholic acid (UDCA) treatment [42].

## 7. Dysbiosis and Liver Cirrhosis

Liver cirrhosis is a severe liver disease characterized by loss of liver cells and irreversible scarring of the liver. It represents an end-stage of all chronic liver diseases. Therefore, it is not surprising that patients with liver cirrhosis have intestinal dysbiosis characterized by significant compositional shifts toward pro-inflammatory bacterial taxa. Gut dysbiosis in liver cirrhosis is accompanied by impaired gut-barrier function, pathological bacterial translocation and “immune exhaustion”. Bacterial components and toxins (defined as endotoxemia) reaching the liver via a disrupted gut barrier accelerate the already existing hepatic injury and increase the systemic inflammatory response. These processes may then induce and promote portal hypertension and other complications of liver cirrhosis like variceal bleeding or ascites [43].

Therapeutic strategies targeting the gut microbiota in liver cirrhosis comprise the use of antibiotics, prebiotics, probiotics, synbiotics, and/or fecal microbiota transplantation [44]. The locally acting antibiotic, rifaximine, was shown to reduce the incidence of hepatic encephalopathy (HE) and to decrease the risk of variceal bleeding [45,46]. 

The role of probiotics in liver cirrhosis is controversially discussed. The evidence supporting a positive effect of prebiotics and probiotics is rather weak due to small and/or heterogeneous studies. However, it was demonstrated that some negative pathophysiologic aspects of liver cirrhosis can be reversed by the use of probiotics. The following beneficial processes were observed: (1) reduction in arterial ammonia concentration; (2) improvement in both overt and minimal hepatic encephalopathy; (3) decrease in bacterial translocation and metabolic endotoxemia; (4) occurrence of anti-inflammatory effects and reduction of pro-inflammatory cytokines such as TNFα; (5) reduction of systemic inflammatory reaction; and (6) improvement in hemodynamic parameters in liver cirrhosis patients [47,48].

Probiotics show promising effects in patients with liver cirrhosis. However, studies investigating their role in the treatment of liver cirrhosis have several limitations. The majority of studies used probiotics for a short period of time (<3 months). A further problem is that different probiotics were tested in different doses; thus, studies show a high likelihood of bias. Further analysis using well-designed randomized controlled trials are required to judge the exact role of probiotic-based therapy in patient with liver cirrhosis.

Not long ago, researchers started investigating the role of fecal microbiota transplantation (FMT) in liver cirrhosis. FMT is a procedure of transplantation of fecal bacteria from a healthy donor into a patient’s gut for restoration of normal colonic flora. This method is gaining popularity because of its high effectivity in the therapy of recurrent *Clostridium difficile* infection [49]. The first controlled study with FMT (used as enema) in patients with liver cirrhosis demonstrated that this method significantly improves cognitive functions of the patients and reduces their hospital stay due to a beneficial shift in gut microbiota composition and an increased microbial diversity [50]. The FMT with antibiotic pretreatment was well tolerated.

## 8. Targeting Gut Microbiota in Hepatocarcinogenesis

Hepatocellular carcinoma (HCC) is the fifth most common cancer and the third most common cause of cancer-related mortality worldwide. There is emerging evidence that the gut microbiota may have influence on the development and progression of this malignancy. The possible mechanisms through which the gut microbiota is implicated in the pathogenesis of HCC involve increased prevalence of pro-inflammatory bacteria due to the intestinal dysbiosis, increased barrier permeability and bacterial translocation from the gut, direct damage of the liver cells by bacterial endotoxins, and microbiota-mediated alterations in bile-acid metabolism. 

The recent study by Ponziani et al. [51] showed significant changes in the gut microbiota profile among patients with HCC. The stool of patients with HCC compared to healthy subjects showed a significant decrease in alpha-diversity. The sequencing of 16S bacterial RNA showed in the stool of HCC patients increased abundance of *Bacteroides*, *Ruminococcus*, *Enterococcus*, *Phascolarctobacterium*, and *Oscillospira* and decreased abundance of *Bifidobacteria* and *Blautia* as compared with liver cirrhosis without HCC. In particular, the deficiency of anti-inflammatory bacteria such as *Bifidobacteria* or *Blautia* can enhance intestinal and liver inflammation and cause the progression of hepatocarcinogenesis. 

All these observations indicate that modulation of the gut microbiota with probiotics in patients with liver cirrhosis and at increased risk for HCC could decrease intestinal permeability and inhibit microbiota-mediated process of carcinogenesis in the liver. In an animal model, Li et al. demonstrated that the probiotic mixture, Prohep, composed of *Lactobacillus rhamnosus* GG (LGG), viable *Escherichia coli* Nissle 1917 (EcN), and heat-inactivated VSL#3 (1:1:1), caused a significant reduction (almost 40%) of HCC growth. The authors postulate that the reduction in the recruitment of T helper 17 (Th17) cells from the gut to the tumor site and decreased plasma levels of pro-angiogenic interleukin 17 (IL-17) may be consequences of the probiotic treatment and responsible for the inhibition of tumor growth [52]. In another animal model of carcinogen-induced hepatocarcinogenesis, the reduction of circulating bacterial LPS using antibiotics prevented HCC growth [53].

These studies indicate that manipulation of the gut microbiota with anti-inflammatory bacteria may prevent bacterial translocation with endotoxin absorption and development of hepatocellular carcinoma in patients who are at risk of developing this malignancy (such as patients with liver cirrhosis or nonalcoholic hepatic steatohepatitis). Further studies in humans should shed more light on the role of probiotics in liver carcinogenesis. 

## 9. Conclusions

In summary, intestinal dysbiosis is observed in many chronic liver diseases (e.g., NAFLD, ALD, immune-mediated liver diseases, liver cirrhosis and hepatic carcinogenesis). There is increasing evidence for an adverse role of intestinal dysbiosis in the pathogenesis and progression of these diseases. Amelioration of the dysbiosis through the use of prebiotics, probiotics, and fecal microbiota transplantation improves the gut-barrier function and appears to be a promising new approach to managing chronic liver diseases.
